# Supplementary material for: Pain and other complications of pelvic mesh: a systematic review of qualitative studies and thematic synthesis of women’s accounts
Source: BMJ Open. 2024 Jun 3;14(6):e085879. doi: 10.1136/bmjopen-2024-085879 (PMC11149113; doi:10.1136/bmjopen-2024-085879)
Supplement: Supplementary data [file bmjopen-2024-085879supp001.pdf]

Supplementary data

Search terms

The search terms used for each database were:

1. quality of life.ab,ti.
2. experience.ab,ti.
3. survey.ab,ti.
4. qualitative.ab,ti.
5. 1 or 2 or 3 or 4
6. Pelvic organ prolapse.mp. or Pelvic Organ Prolapse/
7. Surgical mesh.mp. or Surgical Mesh/
8. 6 and 7
9. Vaginal mesh.mp
10. Transvaginal mesh.mp
11. TVT.mp or transvaginal tape.mp
12. 8 or 9 or 10 or 11
13. 5 and 12

Table: Amalgamated CASP and COREQ quality appraisal

**Domain/item & guide questions / Studies**	***Interviewer/facilitator, training, relationship with interviewees***	***Methodological orientation or theory***	***Method approp-riate to aims?***	***Interview guide***	***Rigour and reflexivity of analysis***	***Derivation of themes***	***Statement of findings***
Brown 2020 [27]	Sole author; nurse & lived experience of mesh. No relationship but experience disclosed to interviewees.	Hermeneutic phenomenology; interpretation of lived experience.	Yes	No detail	Used framework; single coder; respondent validation; some reflexivity.	From data.	Largely descriptive, but met aims.
Cumberlege (chair) 2020 [16]	Independent enquiry. Panel asked questions; written testimonies also used.<br>No relationship with interviewees.	Legal: evidence gathering.	Yes	N/A but full transcript.	N/A: no data analysis.	N/A	Full summary of findings, and women with mesh complications involved in recommendations.
Dibb et al. 2023 [30]	One author; no details of training or relationship with interviewees.	Thematic analysis.	Yes	Some detail	Little detail; some themes are close to question topics; some reflexivity.	From data.	Positive and negative aspects described and extensive use of quotations.
Dunn et al. 2014 [33]	Two researchers; no further detail.	Qualitative description, no inference.	Yes	Two open-ended questions supplied.	Multiple coders & respondent validation; little description of analytic process; no interpretation by design; little reflexivity.	From data.	Describes women's experience in three trajectories.
Huntington et al. 2019 [35]	No interview: women submitted personal accounts.	Inductive: thematic analysis	Yes	Prompts for written account described.	Little description but thematic analysis method used; no mention of reflexivity.	From data.	Answer research questions but without critical analysis.

Izett-Kay et al. 2020 [31]	Free text on postal questionnaire or phone sampling. Some respondents might have been patients of authors.	“Interpretivist” approach; inductive; thematic analysis	Yes	Single question supplied.	Two coders and team discussion; little description of analysis; no reflexivity but mention of possible unconscious bias.	From data.	Findings clearly described; theme titles not very descriptive.
McKinlay & Oxlad 2022 [36]	Written submissions to government enquiry. No relationship.	Deductive and inductive thematic analysis.	Yes	N/A	Detailed description of analysis, using biopsychosocial framework. No reflexivity by design.	From data, then grouped.	Clearly reported with recommendations.
Toye et al. 2023 [32]	Data collection described in related paper; no details of interviewers or possible relationship.	Reflexive thematic analysis.	Yes	Example questions in text, developed with PPI contribution.	Detailed description: multiple coders; PPI involvement in analysis; reflexivity described.	From data.	Full account with supplementary data; interpretation of findings to draw higher level lessons.
Uberoi et al. 2021 [34]	Data collection by trained and experienced authors, avoiding those who knew interviewees.	Deductive and inductive content analysis.	Yes	Interview guide supplied.	Detailed description; multiple coders; no mention of reflexivity.	From data, grouped by interview prompts.	Themes literal rather than latent meanings, perhaps because used prompts as themes.

Key: N/A not application; PPI patient and public involvement
